# Transactions of the Annual Meeting of the Medical Society of the County of Erie

**Published:** 1855-02

**Authors:** 


### ART. IV.—*Transactions of the Annual Meeting of the Medical Society of the County of Erie.*

The annual meeting of the Medical Society of the County of Erie, was held in this city on the 9th of January, at American Hall, commencing at 10 o'clock, A. M.

The *Vice-president*, Dr. James P. White, presiding, the President being absent.

The attendance was large, fifty-one members being present during the session.

Dr. J. C. Lay was admitted to membership with the society. A diploma was presented by Dr. Mackay, from the State Medical Society, as an evidence of his compliance with the laws of the state relative to the admission of foreign practitioners; and the previous action had by this society in admitting him to membership was affirmed.

The *Librarian's Annual Report* was presented by Dr. Samo, containing many valuable suggestions relative to the improvement and preservation of the library; and on motion of Dr. Treat, so much of it as referred to obtaining missing numbers of the Transactions of the State Medical Society was confided to the attention of the Delegates to that Society, and the Librarian was directed to effect an insurance on the library to the amount of three hundred dollars.

The *Treasurer*, Dr. Bailey, reported the receipts during the year $46 50, and the expenditures $63 00, and the balance on hand $8 24.

Dr. Treat, from the *committee* on the purchase of books, reported the purchase for the library of the entire series of the Sydenham Society's publications, thirty-two volumes in all, at the cost of $64 75; and recommended an annual subscription to the Sydenham Society for the purpose of continuing in the regular receipt of its publications. The report was adopted, and six dollars placed in the hands of the librarian to make the subscription proposed.

Dr. Hunt stated that being in attendance at the last meeting of the State Medical Society, he made inquiries relative to the necessity of the contribution of five dollars usually made to the State Society, but withheld at our last annual meeting, and was informed that such contributions were necessary to defray expenses of the said society, (printing, &c,,) and concluded by *moving* that five dollars be voted for the present year, and also a like amount for last year's contribution.

The resolution was adopted.

The *Oration* was then delivered by Dr. Boardman. Subject, "*The Early History of Medicine.*"

At its conclusion, on motion of Dr. Strong, a vote of thanks was tendered the speaker, and a copy of the address requested for preservation in the archives of the society.

The following preamble and resolutions were offered by Dr. Congar:

*Whereas*, The appointment of Health Physician of the port of New York devolves upon its recipient great responsibilities, in promoting the advancement of medical science by the ample opportunities afforded for supplying useful statistics and observations,

*And whereas*, These ends can only be accomplished by placing some one in that position, of such erudition and habits of observation as qualify him for the competent discharge of these peculiar duties,

*And whereas*, Disinterested medical men can best judge of the requisite fitness of the several members of the medical profession for this important place,

*Therefore Resolved*, That We, the Medical Society of the County of Erie, beg most respectfully to urge upon His Excellency Governor Clark, the appointment of Prof. Charles A. Lee to this office, as being in our opinion eminently qualified, by his industrious habits of observation and study, and by his ability as a writer and teacher in the several departments of medical science, to supply the requisite scientific statistics and deductions; and in our belief combining also all the qualifications necessary for the satisfactory performance of the ordinary duties pertaining to that office.

*Resolved*, That a copy of these resolutions be signed by the president and secretary of this society, and transmitted to Gov. Clark through our Sénator, Mr. Putnam, and that he be requested to urge upon the Governor the importance of these considerations and of the appointment indicated.

*On motion* of Dr. Hunt, the foregoing preambles and resolutions were unanimously adopted.

The *Dinner Committee*, through Dr. Baker, reported that arrangements had been made for the social entertainment of the society at the American Hotel, at 4 o'clock, P. M.

Dr. Nelson offered the following resolution:

*Resolved*, That the secretary be authorized to draw out a list of the medical practitioners, to be headed "Medical Practitioners in the County, legally qualified and recognized by the Erie County Medical Society," for the purpose of being inserted in the Buffalo City Directory, on the leaf before the list of medical practitioners as now printed: and that the secretary be further empowered to add, from time to time, the names of practitioners as they become qualified according to law. *Adopted.*

Dr. Storck stated that the reputable German medical practitioners of the city had formed a society for their own benefit, and for their protection against quackery as practiced by unqualified practitioners among the German population: and that the members of the said society were desirous of becoming legalized practitioners, and of uniting with the Erie County Medical Society, and desired information as to the necessary steps to be taken to accomplish these objects.

*On motion* of Dr. Wilcox, a committee was appointed to confer with the German society, and to furnish them such information and assistance as may be in their power to aid them in the accomplishment of the objects desired

Drs. Storck, Nelson, and L. P. Dayton, were appointed such committee.

Dr. Wilcox introduced the following preamble and resolution:

*Whereas*, Doctor John D. Hill in applying for and accepting the appointment of Physician to the Almshouse for a compensation less than the rates adopted by this society, thereby avoiding fair and honorable competition, has forfeited his claims as a member of this society,

*Therefore resolved*, That the society do now proceed to the trial of John D. Hill for infraction of the rules and regulations of this society.

Dr. Wyckoff doubted whether this subject was in order, and the society competent to act upon it at this time, as the matter had been before the society and been acted upon at the special meetings in October last.

The chair decided the matter to be *in order*, and properly before the society at this time.

Dr. Bailey, to test the sense of the meeting upon the subject, *appealed* from the decision of the chair.

The question "shall the decision of the chair be sustained," was put, and the majority of the votes was in favor of the decision; so the chair was sustained.

A lengthy and animated discussion then proceeded upon the preamble and resolution, and upon taking the vote the *ayes* and *nays* were called for and taken with the following result:

*Ayes* 17. *Nays* 22, and the resolution consequently *lost*.

Thereupon Dr. Hawley introduced the following:

*Whereas*, Dr. John D. Hill, in applying for and accepting the appointment of Physician to the Almshouse for a less compensation than the rates adopted by this society, thereby avoiding fair and honorable competition, has forfeited his claims as a member of this society.

*Therefore resolved*, That the said J. D. Hill be and is hereby expelled from the Erie County Medical Society.

An animated and lengthy discussion ensued upon the above, the chief point in the debate and in the difference of individual opinions being, as to the proper course to be pursued in the matter under consideration.

The previous question having been moved, and the *ayes* and *nays* called for, permission was given by the society for Dr. Ham to introduce the following:

*Ordered*, That the president of the society be directed to serve a copy of the foregoing preamble and resolution on Dr. J. D. Hill, and notify the said Hill to appear before this society for trial, at this place, on the day of the present month, and that when we adjourn we adjourn to meet at this place on the day of the present month.

The previous question being again called, the vote was taken upon the original preamble and resolution, by *ayes* and *nays*, and they were adopted by the following vote:

*Ayes* 19. *Nays* 17.

Five of the members who recorded their votes in the negative, did so with the explanation that they so voted for the reason of a want of a conviction of the propriety of so summary proceedings in the case, and not from sympathy with the departure from the action had by the society upon the Tariff of Prices, or a desire to shield the offender from the consequences of such departure therefrom.

Dr. Mixer was appointed *Orator* for the next semi-annual meeting, and Dr. E. P. Smith his substitute.

The society then proceeded to the election of officers for the ensuing year, and made choice of the following gentlemen to the respective offices:

| | |
|---|---|
| Dr. James P. White, . . . | *President.* |
| " Wm. Van Pelt, . . . . | *Vice-president.* |
| " James M. Newman, . . . | *Secretary.* |
| " Samuel G. Bailey, . . . | *Treasurer.* |
| " James B. Samo, . . . | *Librarian.* |

*Primary Board.*

Dr. J. Eastman,
" W. Ring,
" J. S. Hawley.

*Censors.*

| | | | |
|---|---|---|---|
| Dr. F. H. Hamilton, | Exam. in | Anat. Phys. and Surgery. |
| " J. B. Samo, | " | " | Prac. Med. and Obst. |
| " W. Treat, | " | " | Chem. and Pharm. |
| " W. Van Pelt, | " | " | Mat. Med. and Botany. |
| " H. M. Congar, | " | " | Med. Juris. and Pathol. |

The President and Secretary were empowered to make out credentials for such members as would attend as Delegates, the American Medical Association, in May next.

Dr. Hamilton *gave notice*, that at the semi-annual meeting of the society he would move the following amendments to the By-laws:

That the Title be amended by striking out the words "Rules and Regulations" so that it shall hereafter read "and declare the following By-laws for the better government," &c.

That Article Second be amended by substituting for the words "a delegate," the word "delegates."

On motion of Dr. Wilcox it was

*Resolved,* That the society do now adopt the Tariff of Prices as presented by the committee in June last, and as adopted at that time by the society, as By-laws, Rules and Regulations of the society.

Dr. Wilcox *gave notice* that he would move in June next to change the salary of the Health Physician, as fixed by the Tariff of Prices, from one thousand dollars to nine hundred dollars per year.

The Valedictory Address of the President was deferred, on motion, until after the dinner; when the society adjourned until four o'clock.

After a short recess the society assembled in the dining room of the American Hotel, and partook of an ample repast prepared under the directions of the society's committee. After full justice had been done to the good things of "mine host," the cloth was removed, and the President's address, in the absence of that officer, was read by Dr. Bailey.

At its conclusion a vote of thanks was tendered the President for his able address.

After a short time longer pleasantly spent together, the society *adjourned.*

